# Exploring the Factors Triggering Occupational Ethics Risk of Technology Transaction in Chinese Construction Industry

**DOI:** 10.3390/ijerph17041175

**Published:** 2020-02-12

**Authors:** Xun Liu, Sen Lin, Lixing Liu, Fei Qian, Kun Zhang

**Affiliations:** 1 School of Civil Engineering, Suzhou University of Science and Technology, Suzhou 215000, China; vvvidastar@163.com; 2 Department of Architecture and Civil Engineering, City University of Hong Kong, Hong Kong 999077, China; linsenjn@163.com; 3 Institute of Engineering Management, Hohai University, Nanjing 211100, China; qf2019@hhu.edu.cn (F.Q.); dreamerzk@126.com (K.Z.)

**Keywords:** engineering technology transaction, occupational ethics risk, factor analysis, construction industry

## Abstract

The importance of occupational ethics risk considerations during technology transaction in the construction industry is acknowledged. This is particularly in that the industry plays a significant part in a nation’s development. The technology transaction has seen an increase in activity due to massive infrastructure development programmers adopted by governments and increase in external investment. The technology transaction, like any other, is not immune to unethical occupational behavior. This study aims to investigate the source of occupational ethics risk during technology transaction in the Chinese construction industry. A review of literature demonstrated that a number of contextual factors can influence unethical occupational risk practices. In total, 130 engineering practitioners took part in a questionnaire survey to explore the source of occupational ethics risk during the technology transaction in the Chinese construction industry. Firstly, there were 25 factors identified through literature review overall, which were sorted and analyzed. Among the twenty-five factors, three were identified as the most significant factors: Unreasonable incentives for technology trading; poor regulation; and asymmetry of information. Then, through exploratory factor analysis (EPA) method, the twenty-five factors were divided into seven groups: legal environment, industry environment, incompleteness of information, asymmetry of information, difficulty of observation of information, differences between the two sides of cooperation, and incorrect conceptual awareness. This study provided an added dimension to the understanding of occupational ethics risk issues during the technology transaction in the Chinese construction industry. This paper therefore contributes to the list of countries where similar studies have been undertaken.

## 1. Introduction

Technology transaction refers to the acquisition of technology from other legal subjects through trade or economic cooperation by enterprises, groups, or individuals, including patent rights, proprietary technology, and technical services [1,2]. In the era of knowledge economy, technology transaction is of strategic significance to nation, industry, and enterprise [3]. In the current developing stage of China, for example, with the initiative of “The Belt and Road” strategies, enterprises sometimes need to introduce advanced technology from developed countries and to share technologies with enterprises in other developing countries. In these processes, engineering technology transaction must be involved. According to statistics, in recent years, in China’s international technology transactions, proprietary technology transactions account for more than 80% [4], and almost every technology transfer contract contains the content of proprietary technology. In the field of engineering design, a large number of proprietary technical achievements in engineering design come out every year. The China Engineering and Consulting Association publishes the proprietary technologies reviewed by various professions every year, only in the construction field from 2015 to 2018 there are 374 engineering design know-how identified [5], and there are a large number of engineering design know-how holders in the construction industry who have not submitted declarations, plus unexamined engineering design know-how in other industries, and patented technologies. The “Implementation Measures for the Evaluation of Design Know-how” issued by the China Survey and Design Association stipulates that “design know-how can be transferred from the holding unit to other design units for use”, indicating the importance of engineering design know-how trading issues in the engineering design industry.

Engineering design know-how transactions have their own particularities, for example, there exist issues of opportunistic psychology and asymmetry of information during evaluation, identification and review of know-how before transaction, and issues of confidentiality during the technology transaction of engineering design [6,7,8]; in addition, the know-how technology itself involves a large number of transaction prices; and there are also a series of problems such as the continuous updating and improvement of know-how after the transaction. There are limitations of existing technology transactions and related research on know-how for resolving these issues. Thus, occupational ethics risk can be easily brought into technology transaction in construction industry. Engineering technology is the key technical unit that decides the feasibility, quality, duration, and cost of the whole project. Therefore, the success of engineering design know-how transactions is, to a certain extent, related to the whole life of the entire project and even the introduction of engineering design know-how units, to keep order and sustainability of engineering technology transaction, it is particularly important to prevent occupational ethics risk of engineering technology transaction and to avoid the adverse influence caused by occupational ethics risk in engineering technology transaction.

Inherently, interests are the ultimate driving force of engineering technology transactions, and the two parties will weigh the expected profits and transaction costs to determine whether the transaction can be successfully completed. Both sides may reduce their efforts or take irregular behavior in order to improve their own interests, which might result in occupational ethics risk. In this case, technology transaction participants should recognize the factors that might trigger occupational ethics risk and reduce these risks through a series of factors that are easy to practice. Motivation is the key to understand moral or unethical behavior [9,10]. “Why would someone do that and when would they do that? Is motivation individual or universal?” These questions are not easy to answer, and one needs to consider not only the motivation of the parties, but also explore its impact on the transaction. Although there have been theoretical and empirical studies on the causes and consequences of occupational ethics risk in technology transactions [2,3,11], most of the current research only considered single factor or several factors but did not systematically identify risk factors.

In the present study, through identifying and analyzing the factors that lead to occupational ethics risk of engineering technology transaction, the research established the framework of group factors by exploratory factor analysis (EFA), complemented the current theoretical research of occupational ethics risk, and provided a theoretical basis for the further study of occupational ethics risk of engineering technology transaction. Furthermore, by identifying the key risk events of engineering technology transaction, it is helpful to better understand the cause of moral risk of the participants in technical transaction and take necessary corrective measures to prevent unethical behavior, which is helpful to improve the awareness and level of moral risk prevention management of the technical exporter, the introducer, intermediary institution, and, so as to prevent the loss caused by moral risk, lay a foundation for the development of the industry and promote the rapid development of Chinese engineering technology.

## 2. Literature Review

### 2.1. Occupational Ethics Risk in Technical Transaction

The term “occupational ethics risk” came from insurance industry, then it was widely mentioned in the field of insurance market, labor contract, public welfare, health care, loan market, and decision-making. Research on occupational ethics risk is becoming more and more extensive. Economists have extended the scope of occupational ethics risk: For instance, opportunism, deception, and laziness are also included in occupational ethics risk [12,13]. On the basis of the studies of Holmstrom [14], Leggett and Strand [15], Elitzur and Gavious [16], and Kardes [17], the definition of occupational ethics risk in this study is the sum of the acts of economic subjects that harm the interests of others in order to maximize their own interests without completely bearing the consequences of their actions.

Technology transaction is a systematic process of introducing, absorbing, applying, and innovating engineering technology, the participant subjects could be, for example, enterprises, scientific research institutes, government, science and technology intermediaries, legal institutions, or financial institutions [2]. It is an interactive market behavior of economic, technological, social, and other elements. Meanwhile, the subject, market situation, and influencing factors involved in the technical transaction are different, dynamic, and the content and extent of the impact of uncertain events on technology transactions are different. In addition, the risk exists in all links of technical transaction in the form of staged progress of increase [18].

Research on occupational ethics risk in technology transaction mainly focuses on unilateral and bilateral occupational ethics risk based on information asymmetry [19,20,21]. Information asymmetry refers to the fact that one party mastered information that the other party does not. Both sides of the technology transaction have the potential possibility of occupational ethics risk, and because of the particularity of technology, there exist difficulties in quantification and contract. At the same time, for a technology exporter, there is a cost to spread proprietary technology, and the efforts of both sides of the transaction in this process determine the level of the cost [11]. Therefore, in the process of technology promotion, consultation, and training, the technology exporter may have occupational ethics risk behavior to safeguard their own interests. For a technology importer, under the information asymmetry situation, when the exporter is unable to inspect the local industrialization of imported technology, brand maintenance, marketing promotion of the actual efforts of technology importers, technology importers are also prone to occupational ethics risk behavior [22].

### 2.2. Factors Leading to Occupational Ethics Risk in Technology Trading

There are many factors that affect the efficiency of technology transaction and the effectiveness of occupational ethics risk prevention, the technology pricing has always been considered as the core factor. From the perspective of information economics, symmetrical information and asymmetric information conditions are the main research directions of pricing mechanism [23]. Under the condition of symmetrical information, a fixed cost contract is usually the best choice [24,25,26]. While under the condition of asymmetric information, royalties can share the risks of both sides of the transaction, and the information advantages of the participants can also be reflected by variable rates [27,28,29,30,31]. Many studies have shown that occupational ethics risk is easy to occur when pricing mechanism is unreasonable [32,33,34].

Factors that leading to the occupational ethics risk of technical transaction are closely related to the characteristics of the field, for example, seek quick success and instant benefits [35,36], opportunistic psychology [18,30,31,37], asymmetry of information [28,31], poor supervision and punishment [38,39,40], poor absorption capacity of the technology importer [41], great difference in cooperation objectives and expectations of the parties [33,42], poor technical competition intelligence capacity of enterprises [43,44], high uncertainty in technology research and development [3,45], fierce market competition [14,39,46], high cost of know-how [1,22,27], and unreasonable incentive mechanism of technology transaction [47,48].

A total of 25 factors that trigger occupational ethics risk in engineering technology transaction were summarized and identified as shown in Table 1.

Although there has been theoretical and empirical research on the causes and consequences of occupational ethics risk in technology trading, most of the current research only considered single or partial factors but did not systematically identify the risk factors. Therefore, this study systematically identified and analyzed the occupational ethics risk factors of technology transaction, established the framework, and improved the theoretical research of occupational ethics risk, providing a theoretical basis for further study of occupational ethics risk of engineering technology transaction. By identifying the key risk events of occupational ethics risk management of engineering technology transaction, we can better understand the causes of occupational ethics risk of technology transaction participants and take necessary corrective measures to prevent immoral behavior, which is beneficial to improve the consciousness and level of occupational ethics risk prevention and management of various stakeholders, such as technology exporter, introducer, intermediary organization, and thus prevent the loss caused by occupational ethics risk.

## 3. Research Methods

**Figure 1:**
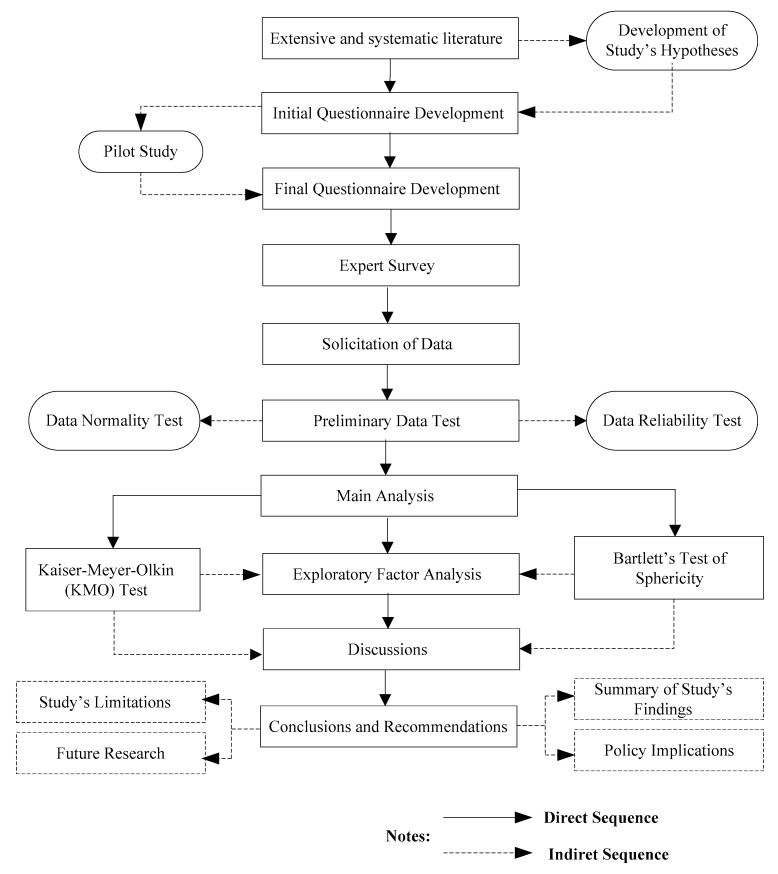
Flowchart of the methodological approach summary.

Following the extensive review of the literature, the study’s questionnaire was first developed (Table S1), and pilot tested with 12 experts from the construction industry for more than 10 years. An expert survey was conducted following the pilot study. The expert survey was conducted via two non-probabilistic sampling approaches known as purposive and snowball sampling approaches. The gathered data were pre-tested to determine their reliability and normality indexes. This constituted the cleaning process of the data. The valid responses were, therefore, examined at the data analysis stage, and the discussions of the results followed this. A summary of the methodological approach of the study is presented in Figure 1 and discussed further in the succeeding section.

In the survey, a questionnaire was developed on the basis of 25 factors, and the respondents were asked to rate the importance of the occupational ethics risk induced by each factor according to the 5-point Likert scale (1 = significantly unimportant; 5 = significant importance). In this study, 12 experts were invited to conduct a preliminary survey of the questionnaire, all of whom were professionals in the Chinese engineering industry for more than 10 years. They summarized the feedback of professionals and revised the questionnaire. The distribution mode of this study is Snowball sampling, which begins with a small population of known individuals and expands the sample by asking those eligible respondents to identify others that should participate in the study. As the sample builds up, enough data are gathered to be useful for research. The first group of respondents were involved in engineering technology transactions, and then they looked for people engaged in related business in their own field as the second group of respondents. Therefore, the similarity of respondents is high, and all of them meet the conditions of the investigation.

During the analysis, descriptive statistical analysis was firstly conducted on the basic data to verify the reliability and validity of the data, and the importance of the role of various factors in triggering the occupational ethics risk of engineering technology transactions was analyzed, and the importance of the factors was ranked. Following the evaluation of the barriers, the variables were factorially analyzed to enable related barriers to be clustered under a unique construct. The EFA tool was used to conduct this analysis due to its ability to factorize a large number of variables into significant fewer constructs [49]. EFA has two key stages: factor rotation and factor extraction. Moreover, other tests ingrained in the EFA tool to check the appropriateness of the data are Bartlett’s test of sphericity and the Kaiser–Meyer–Olkin (KMO) test [50]. To test whether the data are suitable for factor analysis, the Kaiser–Meyer–Olkin (KMO) and Bartlett’s Test were first used. KMO is an index to compare the observed correlation coefficient with the partial correlation coefficient. Given a range of 0 to 1, any KMO value closer to zero connotes a dispersed interrelated pattern of variables, which makes the dataset unsuitable for EFA. Conversely, a KMO value of or closer to 1 represents perfectly compacted interconnection patterns which ensure data suitability for EFA. However, any KMO value 0.5 is satisfactory for EFA to proceed [51,52].The Bartlett’s Test is used to test the homogeneity of variance, a necessary condition for factor analysis. Both KMO and Bartlett’s test of sphericity analyses were conducted to examine the appropriateness of employing EFA technique in this study. A KMO value should be higher than the 0.5 threshold; meanwhile, the significance level of the Bartlett’s test for sphericity should also be small (e.g., *p*-value = 0.000) [40]. Factor extraction determines the initial factor grouping between variables. The methods of factor extraction include principal component method and principal axis factor method [53]. This study used principal component analysis method to identify the grouping of factors. The purpose of factor rotation is to better understand and explain the practical significance of the factor and determine the final value of the grouping. Factor rotation can be orthogonal or oblique. This study adopted the Promax rotation to analyze the data with correlation of components.

## 4. Empirical Results and Data Analysis

### 4.1. Questionnaire Survey

In order to ensure the diversity of the data sources of the questionnaire to improve the applicability of the research results, the survey objects mainly cover three groups, one is the industry with rich experience in technology introduction, the second one is the scholars who have deeply studied the engineering technology transaction, and the third is the legal personnel engaged in technology transaction. A total of 130 available questionnaires were returned completed. Among them, 81% of the respondents have more than 5 years of working experience, and 50% of the respondents have more than 10 years of working experience, which further ensures the quality of the data.

### 4.2. Ranking of Risk Factors

The overall Cronbach’s alpha (Cronbach alpha coefficient) of the factors in this study is 0.850, greater than the recommended threshold value 0.7 [54], which indicates high internal consistency of the questionnaire measurement and data are overall reliable. Table 2 showed the average value and significance level of each ranking factor. The single sample T test was used for validity analysis, when the average value of the factor was greater than 3.00, and the significance level *p*-value was 0.000 less than 0.05, it indicated that the factor was significantly important. This study, based on validity analysis results shown in Table 2, the average value of 20 factors is more than 3, and the significant level *p*-value was less than 0.05, which indicates that these 20 factors are of great importance.

In this study, 25 factors were ranked according to the average value of data. The top five factors were: Unreasonable incentive mechanism of technology transactions, unfavorable supervision, asymmetry of information, lax legal punishment, and excessive cost of default determination. Prioritizing the importance of key factors enables people in business to better understand which areas of ethical risk management are worth preventing, controlling, and prioritizing resources for management. The above five factors will be analyzed in detail.

Unreasonable incentives for technology trading ranked first among all factors. “Unreasonable incentive mechanism” indicates “Administrative monopoly and local protectionism”. Administrative monopoly and local protectionism are major causes of confused contracting. Unreasonable incentive mechanism creates an improper incentive mechanism for government officials. The combining of administrative function and business operation places some government officials in the position of being both judges and players in the construction market [47]. Driven by their own interests, they can intervene unnecessarily in economic activities. Local protectionism, to a great extent, is a form of administrative monopoly [48]. Generally, the more underdeveloped the regions, the more serious the local protectionism. Administrative monopoly has become the biggest institutional bottleneck restricting the development of the construction market. It shows that this factor is the most important factor to cause occupational ethics risk in engineering technology trading. The technology exporter has clear motivation for the technology transaction, mainly including the extension of life cycle of innovative products and technologies, the strategy of global business layout, and the mandatory collateral requirements for foreign direct investment by the governments of the importing countries. In fact, the most important purpose of the technology exporter to the technology transaction is to obtain maximum profit, the way and level of the technology transaction is completely considered from their own interests. Previous research on technology transaction has mainly focused on the incentive mechanism and price clause design in technology transaction contract [22,24,26,55]. It can be seen that the rationality of incentive mechanism in technology transaction contract clause has a great impact on occupational ethics risk.

Poor regulation ranked second among all factors. Due to the characteristics of high investment, high risk, information asymmetry, and uncertainty of engineering technology, evidence is hard to obtain if there is a dispute. Therefore, it is difficult for one actor to supervise the behavior of the other actor. Once the incentive mechanism lost its balance, the benefits do not cover the costs, or, due to other reasons, the actor is likely to break the contract or have opportunistic behavior to damage the rights and interests of the other party. However, the law cannot impose proper sanctions on it, which makes the possibility of occupational ethics risk in the performance of the technical contract extremely great.

Asymmetry of information ranked third among all factors. The asymmetry of information is determined by labor division of labor and specialization. The exogenous information asymmetry is the basis and premise for the generation of technology transaction demand. If the demander has mastered the core technical information owned by the technology supplier and both parties have the common knowledge before signing the contract, the demander will not have the purchase motivation and cannot come to the agreement on technology contract. However, afterwards the endogenous information asymmetry determines the difficulty of performing the technical contract. The behavior of the actor is more operable because it cannot be supervised, and it is easy to cause occupational ethics risk in the performance of the technical contract.

Lax legal punishment ranked fourth among all factors, which is the main cause of occupational ethics risk for contractor. The lack of deterrence and sanctions is the key cause of default in the engineering industry. In theory, imposing major sanctions could greatly reduce corruption. However, the industry’s intellectual-property-related penalties are trivial compared with the huge engineering profits. At the same time, the depth, scope, breadth, and intensity of sanctions are not guaranteed, as corruption and malfeasance by public officials will hide crimes and even divide this information into “national interests”, thereby preventing their disclosure in the public domain.

Excessive cost of default determination ranked fifth among all factors. This has a lot to do with the characteristics of engineering technology itself. Engineering technology refers to the relevant technical knowledge, technical experience, methods, information, or combination of these formed in the design work of the engineering design unit. Its products are reflected in new materials, new equipment, and new technology. For the collection of evidence for breach of contract litigation, the method of product evidence collection is generally adopted. In some large factory processes, the collection of evidence involves the internal structure of the equipment, and the collection of evidence is generally located in the plant where the equipment is located. The uncertainty of whether the factory will cooperate in obtaining evidence has caused the difficulty and high cost of obtaining evidence. The high cost of default identification reduces the cost of occupational ethics risk and connives the occurrence of occupational ethics risk.

### 4.3. Factor Analysis for Underlying Groupings

EFA is a technology that can explore the internal basic structure of multiple observation variables and conduct dimensionality reduction processing. The process of EFA is to seek for a few potential common factors (latent variables) and construct a factor structure so as to maximize the interpretation of the main information of the original variables (observed variables). EFA can be used as the premise of establishing latent variable model or confirmatory factor analysis. This study used exploratory factor analysis to carry on dimension reduction analysis, explored the basic grouping of these 25 factors, and used SPSS 20.0 statistical analysis software to analyze. The general standard for factor analysis is that the sample size is greater than 100, and the ratio of the sample size to the number of variables is greater than 5.00. In this study, the sample size was 130, and the ratio of the sample size to the number of variables was 130/25 = 5.2, so the sample size met the conditions for factor analysis. The factorability of correlation matrix was tested by KMO index (KMO ≥ 0.50) and the test for Bartlett’s sphericity. The KMO value was 0.669, indicating a high degree of common variance among the inducers. The value of the test statistic for Bartlett’s sphericity was large (chi-square = 1804.226) and the *p*-value was 0, suggesting that the correlation coefficient matrix was not an identity matrix. Hence, the data collected were suitable for EFA.

Principal component analysis (PCA) was used to identify the internal groups of factors, and seven factors were extracted, all of which had eigenvalues greater than 1.000. The total variance explained of factors in these seven groups is 62%, which is greater than the recommended threshold value of 60%.

Although orthogonal rotation can easily explain and represent the results of factor analysis, it is often based on the assumption that there is no correlation between molecules, which is often not in line with reality. In addition, even if there is no correlation between factors, the orthogonal state should be verified by confirmatory factor analysis of oblique rotation method. Therefore, this study analyzed the correlation data of composition by using Promax rotation. Results of the Promax factor analysis were shown in Table 3.

**Figure 2:**
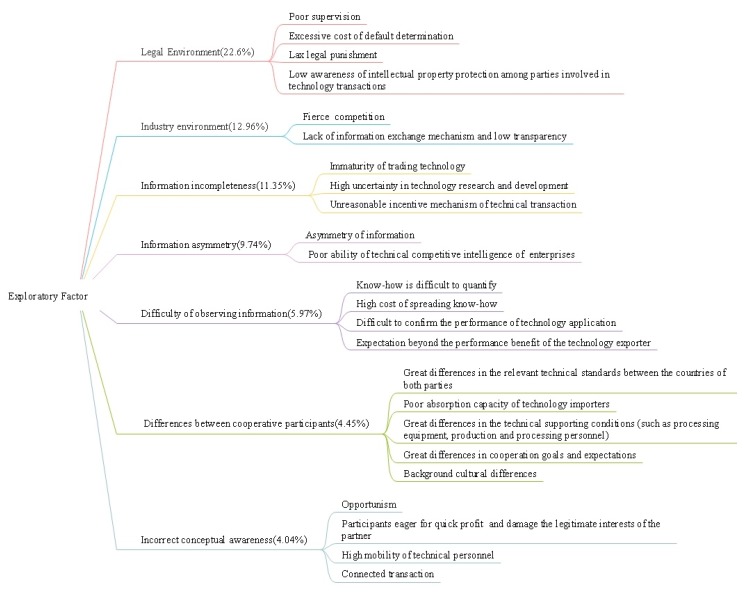
Causal factors of occupational ethics risk.

The extracted factor is named according to the common characteristics of the factors in each group. These 7 groups of factors are named as legal environment, industry environment, information incompleteness, information asymmetry, difficulty of observing information, differences between cooperative participants, incorrect concepts, and consciousness. Figure 2 provides a series of causal factors of occupational ethics risk.

## 5. Discussion and Factors Analysis

### 5.1. Group 1: Legal Environment

The variance explained in the first factor was 22.6%, which included poor supervision, excessive cost of default determination, lax legal punishment, and low awareness of intellectual property protection among parties involved in technology transactions.

At present, the transaction of engineering technology mostly depends on the domestic law of the country where the contract subject is. Immature legal system and the management system such as lax enforcement of law, poor legal environment, and powerless punishment of default behavior could all make less protection of the rights and interests of other relevant parties, which is an important cause of the occupational ethics risk.

Poor supervision: Supervision functions include formal and informal supervision functions. Formal supervision includes political rules, economic rules, and contracts. Effective formal rules and regulations are the premise and basis of technical contract performance. Informal supervision is an informal or unwritten part of a social system structure, which mainly refers to codes of conduct, codes of conduct, and customs. Formal restriction only provides a good institutional background and environment for the performance of technology contract, and effective informal restriction is the key to ensure the performance of technology contract. At present, China’s technology market is manifested as an informal market, with imperfect market subject system, unclear property right definition, imperfect information communication system, and inefficient formal restriction mechanism of contract performance. In the transitional modern Chinese economy, the traditional moral concept and code of conduct have been destroyed to a certain extent, and the new concept and criterion, that is, the informal restriction mechanism has not yet been established. Therefore, on the one hand, the objective environment of technical contract performance is not perfect, on the other hand, the subjective factors of contract performance are not perfect and mature. Due to the lack of effective regulatory mechanism in Chinese technology market, the behavior of breaking contract cannot be effectively supervised, the cost of breach of contract is reduced, which further enhance the emergence of occupational ethics risk.

High cost of safeguarding rights: The high cost of safeguarding rights mainly includes three aspects: (1) Difficult to obtain evidence. The exclusive and invisible characteristics of intellectual property rights brings great secrecy to the infringement. The court and other authorities have strict requirements on the form of evidence, which increases the difficulty of proof and the cost of determining breach of contract. (2) High time cost. The litigation of the patent is generally longer than the other litigation, sometimes the patent is invalid or has no market value while the patent rights protection is successful. (3) High cost. Patent litigation costs notary fees, evidence collection fees and attorney fees.

Lax legal punishment: In practical cases, the general amount of compensation in patent infringement cases is within the scope prescribed by the state, and the court determines the specific amount, but in fact the compensation received by enterprises is often lower than the expenses of litigation.

Weak intellectual property protection awareness of enterprises: (1) Enterprises lack the awareness of legal protection of intellectual property rights, and lack of understanding of international intellectual property protection conventions and domestic intellectual property laws. They are usually less aware of relying occupational legal resources to avoid legal risk when signing technology transactions. (2) Cumbersome procedures, difficulty in investigation and evidence collection, and high cost of rights protection caused by the requirements of laws and regulations will lead to the lack of initiative of rights protection.

### 5.2. Group 2: Industry Environment

The variance explained of the second factor was 12.96%, which included: Fierce competition, lack of information exchange mechanism, and low transparency.

As the mastery of engineering technology directly affects the core competitiveness of engineering design units in the field of engineering design, under the incentive of the market environment, in order to enhance their own competitiveness in the industry, each engineering design unit has contributed to the emergence of engineering design proprietary technology transaction.

Fierce competition: Competition is the guarantee of effective market operation. Excessive or vicious competition destroys the fair competition environment. With the increase of competition, there will be a tendency to deceive, cheat and responsibility shift to harm the interests of other parties so as to achieve competitive advantage. Some engineering enterprises are too eager to obtain the latest innovation technology but underestimate risk, likely bring potential occupational ethics risk. Fierce competition makes enterprises beyond the bottom line of morality, lose their reasonable interests, and form a vicious circle in the industry in long term.

Lack of information exchange mechanism and low transparency: With the increasing competition among industries, the technology and information of similar industry is extremely confidential. If there is technology transaction dispute, a technology importing or exporting company might try to take measures to hide defaults behavior to maintain their market reputation, and sometimes the behavior of one party’s actors in default cannot be disclosed and monitored, which increases the cost of determining the default behavior and encourages the occurrence of occupational ethics risk.

### 5.3. Group 3: Information Incompleteness

The variance explained of the third factor was 11.35%, which included: Immaturity of trading technology, High uncertainty in technology research and development, Unreasonable incentive mechanism of technical transaction.

The incompleteness of information is determined by uncertainty. In the process of technology research and development, the contract parties will face unpredictable changes, and the terms in the contract are not perfect because of the incompleteness of the information. Uncertainty and incompleteness of information will increase the probability of occupational ethics risk.

Immaturity of trading technology: The engineering design proprietary technology was formed on the basis of proprietary technology development, technical tackling, and mature stage. A more mature technology could lead the direction of engineering design, and the easier it is to form monopoly price in the transaction, which also affects its expected profitability to a certain extent. The risk of failure is the greatest in the early stage of research and development of technology. Therefore, before the completion of technology transactions, more risks are faced by the technology supplier than technology demander. The technology supplier transfer risks to the technology demander through technology transfer. In essence, technology transaction is a fair distribution of all the expected benefits between technology supply side and technology demand side, it also represents transfer and distribution of risks.

High uncertainty in technology research and development: Uncertainty in technical cooperation mainly stems from two basic aspects: on the one hand, it depends on the nature and process of science and technology; on the other hand, it depends on the complex change of market demand structure and the uncertainty of business cycle. The degree of uncertainty is determined by the complexity of the problem to be solved and the knowledge and ability of the problem solver. If engineering technology wants to be practical and economical, it needs to go through complex theoretical verification, semi-industrial testing, and industrial testing, which may lead to failure due to the uncertainty of technology research and development.

Unreasonable incentive mechanism of technical transaction: The uncertainty of technology determines the incompleteness of the technology contract. In engineering practice, the production and application of technology are subject to huge uncertainties, it is impossible for the parties to accurately consider all contract terms, and incentive mechanism may not effective to its maximum extend. When there is greater benefit from defaults, it is possible for one of the parties to carry out opportunistic behavior, and thus resulting in occupational ethics risk.

### 5.4. Group 4: Information Asymmetry

The variance explained of the forth factor was 9.74%, which included: Asymmetry of information, and poor ability of technical competitive intelligence of enterprises.

Information asymmetry means that in the process of technology trading, one party has information and knowledge that the other party does not know or cannot verify. In terms of time, asymmetric information may occur either before or after the technical contract is signed. In terms of content, one type is exogenous asymmetric information, which is determined by the characteristics of the technology itself; the other type is endogenous asymmetric information, which refers to the information asymmetry caused by one party’s failure to observe, supervise and verify the behavior of the other party after the technology contract is signed.

Asymmetry of information: Exogenous asymmetric information is the basis of technology transaction demand. If the demander had mastered the core technology and knowledge that suppliers have, then the demander side will not have purchasing motivation, then the endogenous asymmetric information will determine the performance of technology contract. The asymmetry of information is determined by division of labor and specialization. Professionals in one field may know little or nothing about other fields, which provide a huge space for opportunistic behaviors, and easily generate occupational ethics risk.

Enterprises’ poor ability of technical competitive intelligence: If the enterprise’s technical competitive intelligence ability is poor, they may not be able to explore the latest core technologies and patent technology. If the relevant characteristics of patent (ownership, scope, effectiveness, limitations, actual control of the owner) are not fully considered in the transaction, the risk of ownership dispute may be faced during the execution of the transaction and the strategic value of intellectual property will be directly affected, which might store up the occurrence of occupational ethics risk.

### 5.5. Group 5: Difficulty of Observing Information

The variance explained of the fifth factor was 5.97%, which included: Know-how is difficult to quantify, high cost of spreading know-how, difficult to confirm the performance of technology application, expectation beyond the performance benefit of the technology exporter.

Know-how is difficult to quantify: The transaction objects in engineering technology transaction include patent technology and know-how. Unlike patented technology, proprietary technology does not have a legal written form of expression. The core of proprietary technology lies in the minds of technical personnel and needs to be revealed by proprietary technology owners. Therefore, proprietary technology is highly subjective. If the technical personnel provide incomplete, wrong, and outdated technical data, the wrong guidance would cause the product fail to meet the specified requirements. Therefore, it is possible for the technical exporter to carry out opportunistic behavior and unwilling to effectively export the proprietary technology, forming occupational ethics risk.

High cost of spreading know-how: Technology training is one of the most important links in technology trading, and it is also an important way to ensure that technology importers can fully obtain technology. Most of the two sides of technology transactions have both a cooperative and a competitive relationship. In order to maintain the monopoly advantage of technology, most technology exporters are unwilling to transfer core technology but only common technology. Moreover, the dissemination of know-how has a high cost for the technology exporter. In addition, the effect of technology dissemination plays an important role in the success of technological innovation, but it is difficult to observe and prove. Therefore, the high cost of disseminating know-how may cause occupational ethics risk of the technology exporter.

It is difficult to confirm the performance of a technology application, and the expectation beyond the performance benefit of the technology exporter: Due to the particularity of technology products, most of the technical factors, market factors, and other random events that affect technology transactions are not predictable when contracts are negotiated. Moreover, the verifiability of the performance produced by technology transaction also has certain limitations, for example, it will be difficult for the technology exporter to observe if the technology importer falsifies the sales performance to save royalty fee. Whether the technology itself have positive effects in the success of technological innovation is difficult to observe and prove. On the basis of these difficulties, when the expected benefit obtained by the performance of the contract is not enough to generate the appropriate incentive to the parties of the contract, and when the party can put off the default at a low cost, both technical supplier and technical demander may have occupational ethics risk.

### 5.6. Group 6: Differences Between Cooperative Participants

The variance explained of the sixth factor was 4.45%, which included: Great differences in the relevant technical standards between the countries of both parties, poor absorption capacity of technology importers, great differences in the technical supporting conditions (such as processing equipment, production and processing personnel), great differences in cooperation goals and expectations, and background cultural differences.

Great differences in the relevant technical standards between the countries of both parties: Technology-leading countries often set technical barriers through technical regulations and standards, so the imported technology usually cannot adapt well to the existing technical resources of the enterprise, and the profit margin of the imported technology is often lower than the product business of the technology exporter in the initial stage. At the same time, it is possible for personnel to misunderstand the application of imported technology which will affect the accuracy of design and application. Occupational ethics risk can easily arise if trading participants try to take advantage of differences in technical standards.

Poor absorption capacity of technology importers and great differences in technical supporting conditions (processing equipment, production and processing personnel): In general, the industrial base, personnel level, and management methods of the importing country are quite different from those of the exporting country, if the gap is too large and there is limited absorption capacity of technology demanders, the technology suppliers may lose their patience in technical training and in providing the latest core technology. When processing and manufacturing licensed products, it is possible that the design quality requirements of a licensed technology cannot be met due to processing accuracy and other reasons. If the imported technology is a product that has been fully tested in the market, the technology exporter will most likely attribute the problem to the production facilities and quality control of the importing party. The product might also be introduced without full market inspection, in which case, both sides may have disputes and leading to greater losses.

Great differences in cooperation goals and expectations: The purpose of the technical importer side is to introduce technology and to improve their technology development. The motivation of the technology exporter is not to help the importer improve their technical level but to obtain maximum profit by prolonging the life cycle of innovative products and technology and layout their strategy of global business for their own interests. The amount of exported technology is quite limited compared with the amount of their monopolized technology, and most of them are mature standardized technology. The key technologies are always mastered and controlled by a technical supplier side, which determines the interest relationship between technology importer and exporter, in this case, there might be occupational ethics risk occurring.

Background cultural differences: A very important part of technology introduction is to integrate the technology with the existing technical knowledge, equipment, management concept, and other technical resources. Background culture has a fundamental impact on conceptual awareness and thinking mode. Cultural background differences between the two sides mean great conceptual awareness differences, misunderstandings, and communication problems might occur. If there is a dispute between the two sides in the process of technological digestion, communication between a party from a country with lower index of power distance (PDI) with a party from a country with higher PDI could bring negative impact on the party from a country with high PDI, and thus bringing potential occupational ethics risk.

### 5.7. Group 7: Incorrect Conceptual Awareness

The variance explained of the seventh factor was 4.04%, which included: Opportunism, participants eager for quick profit and damage the legitimate interests of the partner, high mobility of technical personnel, and connected transaction.

Opportunism: The actor may pursue wealth under incorrect social value orientation. Conceptual awareness is an informal constraint mechanism, which mainly rely on the actor’s self-control. There will be occupational ethics risk in the performance of the contract if the actor has incorrect conceptual awareness.

Participants eager for quick profit and damage the legitimate interests of the partner: Management concepts of many actors will also bring obstacles to the effective performance of the contract. Lack of long-term cooperation goals and business management methods, they are eager for quick profit and harm the legitimate interests of their partners. If the actor does not have effective self-discipline, the probability of occupational ethics risk could be greatly increased.

High mobility of technical personnel: Technical personnel’s experience play significant role in engineering patented technology or engineering proprietary technology. Personnel mobility or technical disclosure may happen if confidentiality agreements are not concluded.

Connected transaction: Connected transaction plays a positive role in realizing optimal allocation of resources. Connected transaction is harmless to market economy and can save social costs if fairness and integrity principles are followed. However, due to the special relationship between the two parties in connected transactions, there are potential conflicts of interest. If the connected transactions are used to escape debts, transfer funds, withdraw excellent assets, and share confidential technologies illegally, occupational ethics risk is easily caused.

## 6. Conclusions and Recommendations

The present research identified the factors that cause occupational ethics risk in engineering technology transactions and studied the internal relationship between these factors. Results showed that: (1) Among the identified 25 factors, 20 of them play a significant role in the initiation of occupational ethics risk behavior in engineering technology transaction, and among which, the three factors: Unreasonable incentive mechanism of technical transaction, poor regulation, and asymmetry of information were of the greatest relative importance; (2) seven groups were obtained based on EFA to summarize the main areas and key problems that lead to the occupational ethics risk of engineering technology transaction: industry environment, information incompleteness, asymmetry of information, difficulty of observing information, differences between cooperative participants, and incorrect ideology. Results obtained in this paper provide reference information for the study of occupational ethics risk and facilitate the establishment of knowledge system of occupational ethics risk in construction engineering industry.

The following management implications can be derived from this study: (1) With the advancement of economic reforms, technology transaction related systems need to continue to develop while operating. The basic goals of these institutional changes are: Transparency, competition, and institutionalization. Together, they are expected to drive the efficiency, fairness, order, and quality of engineering technology transactions; (2) the unreasonable incentive mechanism of technology transactions is the most important trigger of occupational ethics risk and should be constantly identified and controlled to prevent unethical occupational behaviors of individuals and organizations caused by pressures in various aspects such as market, competition, cost, and quality; (3) The risk management communication between the enterprise and the project needs to be strengthened. The occupational ethics atmosphere of the company has a significant impact on the project’s moral risk management; (4) In order to form a better organizational atmosphere, the company should establish occupational ethical standards and strengthen internal implementation.

This study identifies the key factors that induce occupational ethics risk in technical trading and explores the grouping of factors. However, the relationship among the groups of factors and the relationship with occupational ethics risk behavior have not been thoroughly studied. Future research can measure the occupational ethical performance of engineering technology transactions by identifying a series of indicators, and further explore the influence path of inducing factors on occupational ethical performance. Meanwhile, the empirical analysis data of this study are mainly from China, which has certain limitations for describing foreign cultures. Future research can be done similarly with reference to the methods of this research based on the characteristics facing other countries and the real-world environment. The results of this study provide reference information for occupational ethics risk research in the global construction industry, and help build a global occupational ethics risk knowledge system.

## Figures and Tables

**Table 1:** Factors that trigger occupational ethics risk in engineering technology transaction.

Number	The Factors of the Occupational Ethics Risk
F1	Lack of information exchange mechanism and low transparency
F2	Fierce competition
F3	Information asymmetry
F4	Poor regulation
F5	Excessive cost of default determination
F6	Lax legal punishment
F7	Immaturity of trading technology
F8	High uncertainty in technology research and development
F9	Great differences in the relevant technical standards between the countries of both parties
F10	Poor absorption capacity of technology importers
F11	Great differences in technical supporting conditions (such as processing equipment, production and processing personnel) between the two sides
F12	Unreasonable incentive mechanism of technical transaction
F13	Difficult to confirm the performance of technology application
F14	The performance income of the technology exporter cannot reach the expected level
F15	The terms of the technology transaction contract do not stipulate the related transactions of the partner
F16	Opportunism
F17	Seek quick success and quick profit
F18	High mobility of technical personnel
F19	The support of the leader of the technology importer is too high or too low
F20	Low awareness of intellectual property protection among parties involved in technology transactions
F21	Enterprises’ poor ability of technical competitive intelligence
F22	Know-how is difficult to quantify
F23	High cost of spreading know-how
F24	Big difference in the target of cooperation between transaction parties
F25	Background cultural differences between the two parties

**Table 2:** Ranking of occupational ethics risk factors.

No.	Occupational Ethics Risk Factors	Mean Value	Ranking	*p* Value
F1	Lack of information exchange mechanism and low transparency in the industry	3.2326	7	<0.001
F2	Fierce competition	3.1628	10	<0.001
F3	Asymmetry of information	3.7364	3	<0.001
F4	Poor regulation	3.9147	2	<0.001
F5	Excessive cost of default determination	3.3256	5	<0.001
F6	Lax legal punishment	3.6744	4	<0.001
F7	Immaturity of trading technology	3.0115	19	0.08
F8	High uncertainty in technology research and development	3.155	11	0.005 a
F9	Great differences in the relevant technical standards between the countries of both parties	3.2016	9	<0.001
F10	Poor absorption capacity of technology importers	3.0212	18	<0.001
F11	Great differences in technical supporting conditions (such as processing equipment, production and processing personnel) between the two sides	3.0367	17	<0.001
F12	Unreasonable incentive mechanism of technical transaction	3.9287	1	0.01
F13	Difficult to confirm the performance of technology application.	3.093	13	<0.001
F14	Expectation beyond the performance benefit of the technology exporter	3.155	11	<0.001
F15	The terms of the technology transaction contract do not stipulate the related transactions of the partner	3.062	15	<0.001
F16	Opportunism	2.9504	24	0.09
F17	Participants eager for quick success and quick profit, damage the legitimate interests of the partner for their own immediate interests	3.0698	14	<0.001
F18	High mobility of technical personnel	2.9844	22	<0.001
F19	The support of the leader of the technology importer is too high or too low	3.0465	16	<0.001
F20	Low awareness of intellectual property protection among parties involved in technology transactions	3.2171	8	0.08
F21	Enterprises’ poor ability of technical competitive intelligence	2.9929	21	<0.001
F22	Know-how is difficult to quantify	2.9791	23	0.06
F23	High cost of spreading proprietary technology (know-how)	2.9461	25	<0.001
F24	Great differences in cooperation goals and expectations of both parties	3.2946	6	0.002 a
F25	Background cultural differences between both parties	3.0037	20	0.07 a

^a^ Single-sample t-test was significant at the significance level of 0.05 (bilateral test).

**Table 3:** Analysis results of exploration factor analysis (EFA).

Number	Group 1	Group 2	Group 3	Group 4	Group 5	Group 6	Group 7
F4	0.888						
F5	0.755						
F6	0.723						
F20	−0.646						
F2		0.891					
F1		0.841					
F7			0.807				
F8			0.796				
F12			0.679				
F3				0.895			
F21				0.743			
F22					0.839		
F23					0.793		
F13					0.665		
F14					0.569		
F9						0.813	
F10						0.769	
F11						0.638	
F25						0.607	
F24						0.502	
F16							−0.848
F17							0.731
F18							0.697
F15							−0.519
Eigenvalue	5.658	3.241	2.836	2.435	1.492	1.114	1.009
Variance explained	22.632	12.964	11.345	9.739	5.968	4.454	4.037
Total variance explained	22.632	35.596	46.941	56.680	62.649	67.103	71.140

## Supplementary Materials

The following are available online at https://www.mdpi.com/1660-4601/17/4/1175/s1, Table S1: Questionnaire.
