# Supplementary material for: Exploring the Factors Triggering Occupational Ethics Risk of Technology Transaction in Chinese Construction Industry
Source: Int J Environ Res Public Health. 2020 Feb 12;17(4):1175. doi: 10.3390/ijerph17041175 (PMC7068282; doi:10.3390/ijerph17041175)
Supplement: Supplementary file 1 [file ijerph-17-01175-s001.pdf]

Supplementary Document

Questionnaire

Research on the Causes of Occupational Ethics Risk in Engineering Technology Transfer

Dear interviewee:

This questionnaire was developed to study the causes of moral hazard in engineering technology transfer.

Occupational ethics risk is defined as the behavior of one party that harms the interests of other parties in order to maximize their own interests in economic activities. Through literature review and interviews, we have summarized the factors that **cause occupational ethics risk in engineering technology transactions**. Experience scores the importance of each factor in triggering occupational ethics risk. The numbers 1-5 indicate the importance from low to high **(1 = not at all important, 5 = very important)**.

Thank you again for your support! Good luck with your work!

Part I. Background information of respondents

Gender: _____ (A. Male, B. Female) Years of work: _____ years

Current position: A. Enterprise executive B. Project manager C. General project manager D. Professional and technical personnel E. Other _____ (Please Specify)

Countries involved in the engineering technology trading project: _____ Your education background: _____ (A. Associate degree B. Bachelor degree C. Master degree D. Doctorate degree)

Working institution type: ____ (A. Scientific research institution B. Design institute C. Consulting company D. Processing and manufacturing enterprise E. other ______)

Part II Answer

**Factors triggering Occupational ethics risk for contractors**		**Importance: Low → High**				
		**1**	**2**	**3**	**4**	**5**
F1	Lack of information exchange mechanism and low transparency in the industry					
F2	Fierce competition					
F3	Asymmetry of information					
F4	Poor regulation					
F5	Excessive cost of default determination					
F6	Lax legal punishment					
F7	Immaturity of trading technology					
F8	High uncertainty in technology research and development					
F9	Great differences in the relevant technical standards between the countries of both parties					
F10	Poor absorption capacity of technology importers					
F11	Great differences in technical supporting conditions (such as processing equipment, production and processing personnel) between the two sides					
F12	Unreasonable incentive mechanism of technical transaction					
F13	Difficult to confirm the performance of technology application					
F14	Expectation beyond the performance benefit of the technology exporter					
F15	The terms of the technology transaction contract do not stipulate the related transactions of the partner					
F16	Opportunism					
F17	Participants eager for quick success and quick profit，damage the legitimate interests of the partner for their own immediate interests					
F18	High mobility of technical personnel					
F19	The support of the leader of the technology importer is too high or too low					
F20	Low awareness of intellectual property protection among parties involved in technology transactions					
F21	Enterprises’ poor ability of technical competitive intelligence					
F22	Know-how is difficult to quantify					
F23	High cost of spreading proprietary technology (know-how)					
F24	Great differences in cooperation goals and expectations of both parties					
F25	Background cultural differences between both parties					
